# Catheter ablation of atrial fibrillation—A key role in heart failure therapy?

**DOI:** 10.1002/clc.23150

**Published:** 2019-02-12

**Authors:** Beatriz Tose Costa Paiva, Thomas H. Fischer, Johannes Brachmann, Sonia Busch

**Affiliations:** ^1^ II. Medizinische Klinik, Kardiologie, Angiologie, Pneumologie Klinikum Coburg Coburg Germany

## Abstract

Atrial fibrillation (AF) and heart failure (HF) are epidemic cardiac diseases and are often detected in the same patient. Recent evidence suggests that this is not a mere coincidence but that the strategy of AF treatment may impact HF development. This review comprehensively summarizes current trial data on rhythm and rate control strategies in atrial fibrillation with a special focus on catheter ablation of AF in HF patients. For a long time**,** rate and rhythm control strategies for AF have been regarded as equal regarding long term mortality. Decision making has been based on the symptoms of patients. Current trials, however, show that the treatment strategy of AF and its effectiveness may significantly impact survival of HF patients. The benefits of rhythm control in HF patients may have been masked by side effects of antiarrhythmic drugs. If rhythm control, however, is achieved by catheter ablation, a reduction of HF related mortality can be observed. As catheter ablation of AF may reduce mortality in HF patients, AF ablation should be preferred over medical treatment in HF patients. In general, HF patients may profit most from rigorous AF treatment.

## 1 PREVALENCE AND RISK FACTORS OF ATRIAL FIBRILLATION

Atrial Fibrillation (AF) is by far the most common sustained arrhythmia and one of the most challenging ones to treat. By the year 2030, there will be an estimated 14‐17 million patients with AF in the European Union.^1^ It is a worldwide leading cause of hospitalizations and cardiovascular death accounting for 35% of all arrhythmia‐related hospital admissions.^2^ Without appropriate therapy, patients are confronted with a 1.5‐1.9‐fold increased risk for death as well as a 5‐fold increased risk for stroke and thromboembolic events.^3^, ^4^

As AF is a heterogeneous and multifactorial rhythm disorder, different clinical presentations from self‐limiting paroxysmal AF to permanent AF are observed. Up to date, the mechanisms of sustained AF and the nature of arrhythmogenic substrates are still a matter of debate and inter‐individual differences between patients most likely account for inconsistencies.^5^

Importantly, recent longitudinal data from the Framingham Heart Study^6^ highlighted the fact that the risk factor burden and the existence of multiple morbidities have a crucial role in the lifetime risk of atrial fibrillation. Participants with at least one elevated risk factor were confronted with a lifetime AF risk as high as 37.8%.^6^ Of note, diabetes mellitus seems to be a major factor for the development of AF. Patients with diabetes mellitus were shown to have a lifetime risk of AF of approximately 40%.^7^ This could be explained by the strong relationship between autonomic dysfunction in diabetic patients and AF.^8^ In line with this, diabetes has been shown to be associated with an increased risk of stroke, even in younger patients.^9^ Other less well‐studied risk factors, such as chronic obstructive pulmonary disease, are also linked to AF,^10^ which is probably driven by systemic inflammation.^11^

## 2 ATRIAL FIBRILLATION AND HEART FAILURE

Currently, an important and increasingly concerning topic is the treatment of AF in patients with heart failure (HF), which despite recent advances in HF‐therapy still continues to be a high‐mortality disease.^12^, ^13^, ^14^

The correlation between both entities was first described in 1937, when Paul Dudley White stated: “Since auricular fibrillation so often complicates very serious heart disease, its occurrence may precipitate heart failure or even death, unless successful therapy is quickly instituted.” How wise this statement was, however, could only be proven in the last couple of years. Indeed, both diseases significantly interplay. Whenever AF and HF coincide, high morbidity and mortality rates are encountered. The incidence of AF and HF is estimated to be around 1%‐2% in adults^15^ and the prevalence of both diseases increases with age. HF is encountered in more than 10% of over 70‐year‐olds^14^ and AF in nearly 10% in the octogenarian population.^16^ Interestingly, up to 50% of patients with HF suffer from AF.^2^, ^16^ Furthermore, the severity of HF facilitates the occurrence of AF. HF patients classified as New York Heart Association (NYHA) I show an AF prevalence of <5%, while patients with NYHA IV symptoms have an AF prevalence of up to 50%.^2^, ^17^

On the other hand, AF can lead to tachycardia induced cardiomyopathy (TIC) and subsequently HF. TIC is frequently underdiagnosed, which can easily lead to the misdiagnosis of idiopathic left ventricular (LV) systolic dysfunction. It has been reported that 58%‐88%^14^ of cases of idiopathic LV systolic dysfunction can be causally linked to AF. The mechanism responsible for the AF related deterioration of LV‐function is rapid and/or irregular ventricular response and neuro‐humoral activation by altered hemodynamics. AF can further lead to a decrease in cardiac output, blood pressure, exercise capacity, and pulmonary congestion—all manifestations of HF.^14^ In line with this, retrospective analyses from the Studies of Left Ventricular Dysfunction (SOLVD) trials demonstrated increased risk for mortality and HF progression in AF patients with asymptomatic and symptomatic LV dysfunction compared to patients in SR.^18^

As TIC is often promptly reversible within a short‐time period after successful treatment of the underlying arrhythmia, recognizing this condition and offering an early appropriate therapy of the underlying pathology is primordial to improve outcomes. In this context, late gadolinium enhanced cardiac magnetic resonance (CMR) imaging identifies irreversible structural change and may predict incomplete recovery of LV function. In the absence of scar, ventricular function normalizes with a high probability following the restoration of sinus rhythm.^19^ Furthermore, LV scar extension has been shown as independent predictor for atrial fibrillation and major adverse cardiac and cerebrovascular events.^20^, ^21^

Apart from TIC, restoration and maintenance of sinus rhythm (SR) in AF patients with HF in general is an approach to decrease symptoms. Upon the reestablishment of SR, HF patients potentially benefit from atrial contraction improving diastolic filling and the restoration of atrio‐ventricular sequential activation. Conclusively, a relief of HF related symptoms was observed upon cardiac rhythm control.^18^, ^22^ However, until recently, randomized trials failed to prove that the hemodynamic benefits of rhythm control strategies translate into decreased mortality.

## 3 PHARMACOLOGICAL RHYTHM VS RATE CONTROL IN HEART FAILURE PATIENTS

Rhythm and rate control strategies are the cornerstone in the treatment of AF. Both strategies aim at improving symptoms.

Several important studies have assessed the outcomes of pharmacological rhythm control in AF patients additionally suffering from heart failure with reduced ejection fraction (HFrEF). In the Danish Investigators of Arrhythmia and Mortality on Dofetilide in Congestive Heart Failure (DIAMOND‐CHF) trial,^23^ published in 2001, 1518 patients were randomized to receive either dofetilide (n = 762) or placebo (n = 758). Study data revealed that 65% of patients randomized to dofetilide therapy were in SR compared to 30% of patients in the placebo group. The dofetilide arm was associated with less frequent hospitalizations due to AF (*P* < 0.001; hazard ratio 0.75; 95% confidence interval, 0.63‐0.89). However, no significant difference in overall mortality was observed between both groups (311 patients = 41% in dofetilide arm vs 317 = 42% in the placebo group).^23^, ^24^, ^25^

Moreover, another large multicenter randomized trial published in 2008 (Atrial Fibrillation and Congestive Heart Failure trial, AF‐CHF)^26^ compared mortality between rhythm and rate control treatment strategies in 1376 AF patients with left ventricular ejection fraction (LVEF) <35% and symptoms of congestive HF. Primary endpoint was the time to cardiovascular death. Of the study group, 682 patients were randomized to rhythm control group vs 694 to rate control group. Antiarrhythmic drugs (AAD) like amiodarone, sotalol and dofetilide as well as electrical cardioversion were used as rhythm control strategy. In the rate control‐arm, beta‐blockers and cardiac glycosides were administered. In line with previous studies, cardiovascular death rate was similar in both groups (27% in rhythm control vs 25% under rate control, *P* = 0.59) after a mean follow‐up period of 37 months. Again, overall mortality did not differ either (32% vs 33%, *P* = 0.68). Patients on rhythm control, however, had to be hospitalized more frequently in the first year (46% vs 39%, *P* = 0.001), which can be attributed to the need of repeated electrical cardioversion (59% vs 9%, *P* < 0.001) and antiarrhythmic therapy adjustment. In this study, the percentage of SR in the rhythm control group was relatively high (70%‐80%) but with a considerable number of patients on amiodarone regimen.^26^ In total, 82% of patients in the rhythm control arm received amiodarone vs 7% in the rate control group.

These results raised doubts about a valuable benefit of rhythm over rate control strategies in patients with co‐existing AF and HFrEF. The fact that rhythm control strategies did not translate into an improved outcome despite obvious hemodynamic benefits seems to be surprising at first sight. However, the benefits of rhythm control could have been masked by adverse events and side effects caused by the AADs used in these studies.^26^, ^27^ In other words: AAD related adverse effects may have neutralized the potential prognostic benefits of maintaining SR.^26^ Indeed, amiodarone therapy, the AAD most often used in patients with HFrEF, has been associated with considerable discontinuation rates due to severe side effects and higher rates of noncardiovascular death.^24^, ^28^, ^29^

Corroborating this hypothesis, regression analyses from the Atrial Fibrillation Follow‐up Investigation of Rhythm Management (AFFIRM) Study showed an association between SR maintenance and mortality risk reduction (hazard ratio = 0.53), while antiarrhythmic therapy was associated with a high mortality rate (hazard ratio = 1.49).^30^ Therefore, an improved survival rate may potentially be expected if SR could be re‐established with therapeutic measures avoiding long‐term AAD treatment.^30^, ^31^

## 4 ATRIAL FIBRILLATION CATHETER ABLATION IN PATIENTS WITH HEART FAILURE

### 4.1 Pulmonary vein isolation a well‐established AF therapy

As pulmonary vein (PV) triggers were encountered in up to 91% of patients regardless of the clinical type of AF,^5^ the elimination of triggers near and from the PVs has been widely accepted to be the cornerstone of catheter ablation of AF. Pulmonary vein isolation (PVI) has evolved over the past decades and incorporated exciting technological innovations providing patients with symptomatic AF a safe and effective alternative to medical therapy. It is recommended as a first‐line therapy in current guidelines from the European Society of Cardiology,^1^ the American Heart Association/American College of Cardiology^32^ and the Heart Rhythm Society^32^, ^33^ for the treatment of symptomatic patients with AF. The level of evidence/class of recommendation is high (IA and IIa‐B) for patients with symptomatic paroxysmal and persistent AF, respectively, who prefer catheter ablation over medical therapy or who are refractory or intolerant to the antiarrhythmic medication.^1^, ^32^, ^34^, ^35^

Although up to 50% of patients suffer an early recurrence after PVI within 3 months after the procedure, up to half of patients with early recurrence remain AF‐free in a long‐term follow‐up.^33^ Late recurrence more than 3 months after ablation occurs in 25%‐40% of cases depending on the population of AF patients and the ratio of persistent to paroxysmal AF.^33^ Overall, the efficacy of PVI is significantly higher than chronic treatment with AADs.^27^, ^33^, ^36^

### 4.2 AF catheter ablation in heart failure

Currently, heart failure guidelines recommend considering AF ablation if a tachycardiomyopathy is suspected but the level of evidence is relatively low (recommendation class IIa, level of evidence C).^37^ This, however, may change in the near future as the body of evidence for AF playing an important role in the development of HF is steadily growing.

Several observational, retrospective single center studies in AF patients with HFrEF already suggested superiority of catheter ablation regarding LVEF improvement, quality of life (QoL), and functional capacity (Minnesota Living with Heart Failure Questionnaire, MLHFQ) over medical management^38^ and subsequently triggered several randomized trials investigating this important issue.

An early small randomized study published by MacDonald et al^39^ in 2011 (Radiofrequency ablation for persistent atrial fibrillation in patients with advanced heart failure and severe LV systolic dysfunction: a randomized controlled trial [RCT]), however, did not show benefits from AF ablation. Investigators analyzed 41 patients with persistent AF and HFrEF (NYHA II‐IV), randomly assigned to ablation vs clinical management, and did not detect differences in terms of LVEF, QoL, functional capacity or N‐terminal probrain natriuretic peptide levels. The non‐superiority of the ablation group in this case, when compared to the other studies, could be attributed to a high rate of complications related to procedure (15%) and a low ablation efficacy as half of the patients presented AF recurrence at 6‐month follow‐up.^25^

The CAMERA‐MRI study (Catheter Ablation vs Medical Control in Atrial Fibrillation and Systolic Dysfunction) yielded much more promising results.^40^ This study, published in 2017, was a multicenter, randomized clinical trial enrolling 68 patients with persistent AF and a reduced LVEF of 45% or lower due to idiopathic cardiomyopathy. After optimization of rate control, patients were submitted to CMR to assess LVEF and late gadolinium enhancement (indicative of ventricular fibrosis). Afterwards, the patients were randomized to either ablation (n = 33) or medical therapy (n = 33). AF ablation in this study routinely included PVI as well as an adjunctive posterior wall isolation. A loop recorder was implanted in order to assess the AF burden. The assessment of the rate control treatment was done by serial Holter analyses. The primary endpoint was LVEF improvement quantified by CMR at 6‐month follow‐up. Indeed, the results revealed a benefit for patients undergoing ablation, such as improvement in absolute LVEF (10.7%; *P* = 0.007) and normalization of LVEF at 6 months (73% vs 29%; *P* = 0.0093). The AF burden after ablation was 1.6 ± 5.0% at 6 months. Furthermore, the absence of ventricular late gadolinium enhancement on CMR imaging was identified as independent predictor of LVEF improvement upon AF ablation.^40^

Another important trial suggesting benefits of ablation in HF patients suffering from AF was AATAC (Ablation vs Amiodarone for Treatment of Persistent Atrial Fibrillation in Patients with Congestive Heart Failure and an Implanted Device), published in 2016.^2^, ^22^ It was an open‐label, randomized and multicenter trial. The study enrolled patients with persistent AF, dual chamber implantable cardioverter defibrillator (ICD) or cardiac resynchronization therapy defibrillator (CRT‐D), NYHA II or III and LVEF <40%. Patients were randomized to catheter ablation (n = 102) vs medical rhythm control with amiodarone (n = 101). The primary endpoint was AF recurrence. Mortality and hospitalization were also assessed as secondary endpoints. After a 24‐month follow‐up, 71 patients (70%) of ablation group were free from AF. In contrast to that, only 34% (*P* < 0.001) were free from AF recurrence in the conservative rhythm control group (amiodarone). Importantly, an improvement of LVEF (*P* = 0.02), QoL (MLHFQ score, *P* = 0.04), and exercise performance by 6‐minute walk distance (6MWD, *P* = 0.02) could also be detected in the ablation group compared to medical treatment. Furthermore, patients undergoing ablation had less hospitalizations compared to patients on amiodarone‐regimen (31% vs 57%, *P* < 0.001, making up for a relative risk reduction of 45%). Although even the mortality rate formally was significantly lower in the ablation arm (8% vs 18%; *P* = 0.037), the event rate was low and the study had not been powered for mortality.^28^, ^41^

In line with this, the CAMTAF (Catheter Ablation vs Medical Treatment of AF in Heart Failure) trial, published in 2014, showed better outcomes with improvement of LVEF, QoL, and exercise capacity after catheter ablation in patients with persistent atrial fibrillation and HFrEF in comparison with pharmacological rate control. It was a randomized trial enrolling 26 patients in the ablation arm and 24 patients for rate control. Freedom from AF was seen in 81% at 6 months after PVI. LVEF at 6 months was 40 ± 12% vs 31 ± 13% favoring ablation group (*P* = 0.015).^25^, ^42^

On the other hand, the results of the CABANA trial (Catheter Ablation vs Antiarrhythmic Drug Therapy in Atrial Fibrillation), presented in 2018, showed no superiority between catheter ablation and medical therapy for new onset AF patients or previously untreated patients regarding cardiovascular outcomes in a 5‐year follow‐up. Patients were randomized to ablation arm (n = 1108) vs medical management (n = 1096). Standard PVI was the approach of choice in the ablation arm. The primary outcome of death, disabling stroke, serious bleeding or cardiac arrest at 5 years in ablation vs drug therapy group was 8% vs 9.2% (hazard ratio 0.86, 95% confidence interval 0.65‐1.15, *P* = 0.3). Although the inclusion criteria were (a) age older than 65 years or (b) at least one of several risk factors (hypertension, diabetes, congestive HF, prior stroke, left atrium size >50 mm, vascular disease, LVEF <35%), HF patients were not well represented in this trial. Only 9% of the patients had cardiomyopathy and 15% chronic HF.^43^

This gap has recently been closed by a large, multicenter RCT. The CASTLE‐AF Study (Catheter Ablation vs Standard Conventional Therapy in Patients with Left Ventricular Dysfunction and Atrial Fibrillation) for the first time compared catheter ablation with medical drug therapy (rate or rhythm control) in a large group of patients with HF and AF. At 33 centers in the United States, Australia and Europe (17 in Germany), 387 patients with either paroxysmal or persistent AF, chronic HF (NYHA II‐IV) and a LVEF of <35% were randomized (179 patients on ablation arm, 184 patients on medical therapy arm). The medical rhythm control approach was done with amiodarone in about 30% of patients. Importantly, catheter ablation significantly reduced AF burden. Since all patients included in the study had an ICD or a CRT‐D device, the time in SR could be exactly quantified and was documented to be 63.1% vs 21.7% favoring the PVI‐arm. The primary endpoint, hospitalization for worsening HF or death, occurred in a median follow‐up of 37.8 months in 28.5% of catheter ablation patients vs 44.6% on drug therapy (*P* = 0.007). The number of deaths after ablation was also lower than on drug therapy (13.4% vs 25% of patients, *P* = 0.01). Benefits were due to a reduced number of hospitalizations for acute worsening of HF (20.7% vs 35.9%, hazard ratio 0.56, 0.37‐0.83), and a decreased number of cardiovascular deaths (11.2% vs 22.3%; hazard ratio 0.49; 0.29‐0.84). Furthermore, there was an improvement in LVEF (median absolute increase at 60‐month 8.0% in the ablation group vs 0.2% in the medical management arm; *P* = 0.005).^44^

Of note, the Castle AF study emphasized the role of continuous rhythm monitoring for quantifying AF burden and predicting cardiac events. In line with this, a reduction of AF events, strokes, and hospitalization by remote monitoring in heart failure patients was demonstrated in the TELECART Study.^45^

Convincingly, a currently published meta‐analysis (2018) of RCTs on atrial fibrillation ablation in patients with HFrEF^41^ analyzed six current RCTs (including AATAC, CASTLE‐AF, and CAMERA‐MRI) and showed that ablation in a selected population significantly improved LVEF, QoL and functional capacity (6MWD). The benefit of ablation (reduction of HF related hospitalizations and overall mortality) was attributed to a reduction of AF burden. The complication rate of catheter ablation was similar to the population with normal heart function.^41^

## 5 ADJUVANT AND FUTURE THERAPIES IN AF AND HF PATIENTS

In addition to catheter ablation of AF, adjuvant therapies may also be considered. Recent advances in the field of cardiac resynchronization therapy like multipolar pacing have shown promising results reducing cardiac mortality, hospitalization rate, and AF burden.^46^, ^47^

In addition, catheter ablation results might be improved by novel therapies. Selectively targeting microRNAs might influence cardiac electrical and fibrotic remodeling after AF ablation.^48^

As inflammation plays an important role in the process leading to AF recurrence after ablation, therapies modulating the inflammatory response such as corticosteroids or colchicine have been proposed.^49^, ^50^ Recently, oral antioxidant treatment has shown to significantly lower inflammation markers but failed to proof a clinical effect in terms of AF recurrence.^51^

## 6 CONCLUSIONS

Currently available data, particularly the CASTLE‐AF trial, clearly suggest that it seems appropriate to advise HF patients with AF to undergo catheter ablation treatment. It can be safely performed and is highly efficient as rhythm control strategy for HF patients with AF. Most importantly, catheter ablation has proven beneficial influences on LV function and overall mortality compared to drug therapy.

Future studies like the RAFT‐AF study (Randomized Ablation Based Atrial Fibrillation Rhythm Control vs Rate Control Trial in Patients with Heart Failure and High Burden Atrial Fibrillation, 2011‐2020) will certainly further expand our knowledge about AF ablation in HF patients with the aim of further improving clinical practice.
